# Synergistic target network construction and dynamic simulation analysis based on a prospective systems pharmacology strategy

**DOI:** 10.1097/MD.0000000000045369

**Published:** 2025-10-24

**Authors:** Hao Li, Ziyu Zheng, Gengyao Zhen, Ke Tang, Haiyan Fang, Jingzhen Wang, Xinli Liu, Deqiang Zhu

**Affiliations:** a Shandong Provincial Key Laboratory of Microbial Engineering, School of Bioengineering, Qilu University of Technology, Shandong Academy of Sciences, Jinan, China; b State Key Laboratory of Biobased Material and Green Papermaking, Qilu University of Technology, Shandong Academy of Sciences, Jinan, China.

**Keywords:** glucose homeostasis, molecular docking, molecular dynamics simulations, network pharmacology, Sang Huang, type 2 diabetes mellitus

## Abstract

Type 2 diabetes mellitus (T2DM) is a chronic metabolic disorder characterized by insulin resistance, low-grade chronic inflammation, and insufficient insulin secretion, influenced by genetic predisposition and detrimental lifestyle choices. It leads to severe complications that significantly impair quality of life. Sang Huang, a rare and valuable medicinal fungus, has potential therapeutic value for T2DM, but its mechanisms remain underexplored. This study utilized network pharmacology to investigate Sang Huang therapeutic potential in T2DM, validated core targets via molecular docking and molecular dynamics simulations, and elucidated its mechanisms. Results demonstrated that estradiol dipropionate (EDP), a key component of Sang Huang, exerted anti-T2DM effects via pathways such as PI3K–Akt. Upon metabolism to estradiol, EDP activated estrogen receptors, triggering the PI3K–AKT1 signaling cascade, which regulates the phosphorylation of FoxO1, GSK3β, and mTORC1. This enhanced glucose–lipid metabolism; improved insulin sensitivity; and preserved β-cell function in the liver, skeletal muscle, and adipose tissue. Additionally, EDP promoted GLUT4 expression and membrane translocation via the AMPK pathway, accelerating glucose uptake and restoring glycemic homeostasis. Molecular docking further confirmed strong binding affinities between 5 active components and these core targets. Molecular dynamic simulations supported the stability of these interactions. Through this study, we have screened the relevant information of Sang Huang active ingredients and preliminarily predicted its potential targets and pathways for anti-T2DM, but further experimental verification is needed. The results of this study prospectively suggest the potential value of Sang Huang in the treatment of T2DM.

## 1. Introduction

Type 2 diabetes mellitus (T2DM) is a chronic metabolic disorder characterized by insulin resistance and a gradual decline in pancreatic β-cell activity. The core pathology of this condition involves reduced sensitivity to insulin and a relative insulin deficiency, ultimately disrupting glucose homeostasis.^[1]^ As the predominant form of diabetes globally, including over 90% of all cases, T2DM has witnessed a marked surge in prevalence over recent decades. According to the 2021 report by the International Diabetes Federation, approximately 537 million adults worldwide are affected by diabetes, with T2DM being the dominant type. Its incidence is accelerating in low-income countries and among younger demographics, highlighting it as a critical public health challenge in the 21st century.^[2]^ The onset of T2DM stems from a combination of genetic predisposition and environmental factors, including obesity, sedentary lifestyles, and high-calorie diets. Chronic hyperglycemia leads to several complications, such as cardiovascular diseases, diabetic nephropathy, retinopathy, and neuropathy, compromising patients’ quality of life, and imposing considerable socioeconomic burdens.^[3]^Furthermore, T2DM is characterized by chronic low-grade inflammation, which serves as the basis for the failure of β-cells in patients with T2DM to secrete adequate insulin during chronic inflammatory processes.^[4]^ A study has demonstrated that patients with T2DM complicated by diabetic kidney disease exhibit significantly higher levels of the Systemic Immune-Inflammation Index compared to those without diabetic kidney disease.^[2]^ It is well-known that hyperglycemia causes metabolic disorders since it triggers “aberrant” pathways that promote oxidative stress in human tissues. The adipose tissue is metabolically active, containing macrophages and stromal cells in addition to adipocytes. These additional cells produce adipokines to regulate carbohydrate metabolism and its sensitivity to insulin that finally favors inflammation and hyperglycemia processes.^[5]^

Despite advancements in current therapeutic modalities, including lifestyle interventions, oral hypoglycemic agents, and insulin replacement therapy, challenges persist in achieving optimal glycemic control, controlling drug side effects, and addressing complications in specific patient cohorts.^[6]^ Thus, elucidating the molecular mechanisms underlying T2DM, enhancing personalized treatment strategies, and identifying novel therapeutic targets are essential in metabolic disease research efforts.

Emerging evidence suggests that β-glucans derived from edible mushrooms, such as *Ganoderma lucidum* (Lingzhi), *Lentinus edodes* (Shiitake), and *Grifola frondosa* (Maitake), enhance glucose uptake in peripheral tissues by activating the AMPK signaling pathway and improve insulin resistance by modulating the gut microbiota.^[7]^ Furthermore, polyphenols from *Hericium erinaceus* (Lion Mane) exhibit α-glucosidase inhibitory activity, thereby delaying carbohydrate absorption.^[8]^ Notably, the synergistic actions of edible mushrooms may alleviate diabetic complications through multi-target mechanisms, as demonstrated by the renoprotective benefits of *Tremella fuciformis* (Snow Fungus) polysaccharides in diabetic nephropathy.^[9]^

Sang Huang, a medicinal fungus formerly categorized within the genus *Phellinus* but recently reclassified under the genus *Sanghuangporus* due to taxonomic revisions,^[10]^ has a long-standing significance in East Asian traditional medicine. First documented in Shennong Classic of Materia Medica (Shennong Bencao Jing), Sang Huang is celebrated for its ability to “nourish the 5 viscera, regulate gastrointestinal qi, detoxify, and staunch bleeding.”^[11]^ Modern pharmacological studies have revealed that Sang Huang is abundant in bioactive compounds, including polysaccharides, flavonoids, triterpenoids, and sterols, conferring antitumor, immunomodulatory, antioxidant, and anti-inflammatory properties. Its potent efficacy in inhibiting tumor cell proliferation, inducing apoptosis, and enhancing chemosensitivity has positioned it as a key player in the search for natural anticancer drugs.^[12]^

The role of Sang Huang in T2DM intervention has rarely been reported. In this investigation, we used network pharmacology and bioinformatics methodologies to prospectively predict and analyze the potential mechanisms underlying Sang Huang therapeutic effects on T2DM, aiming to facilitate future research initiatives.

## 2. Data sources and research methods

### 2.1. Compilation and target prediction of chemical constituents in Sang Huang

Chemical constituents of Sang Huang were retrieved from the BATMAN-TCM database (http://bionet.ncpsb.org.cn/batman-tcm/) using the search terms “Sang Huang,” “*Phellinus igniarius*,” and “Sanghuangporus.” The search results for Sang Huang and *P igniarius* exhibited considerable overlap. Consequently, the subsequent analyses were based on the data obtained by prioritizing “Sang Huang” as the primary search term. High-confidence protein targets associated with Sang Huang were carefully selected as potential targets, with thorough removal of redundant or invalid entries. These curated targets were standardized to human gene names using UniProt (https://www.uniprot.org/), ensuring the exclusion of non-human genes.

### 2.2. Acquisition of targets related to T2DM

T2DM-related targets were comprehensively compiled by querying the GeneCards (https://www.genecards.org/) and OMIM (https://omim.org/) databases using the keyword “type 2 diabetes mellitus.” The retrieved targets from both databases were consolidated into a spreadsheet, with duplicates removed. These entries were then standardized using UniProt to produce a definitive list of disease-associated targets.

### 2.3. Prediction of Sang Huang–T2DM target interactions

The drug component targets and disease targets were cross-referenced to identify overlapping genes through Venn diagram analysis. A “drug–component–target” interaction network was constructed using Cytoscape 3.8.2.2 (Cytoscape Consortium , La Jolla).

### 2.4. Construction of protein–protein interaction (PPI) network

To further investigate the protein interactions involved in Sang Huang treatment of T2DM, we uploaded the intersection genes to the STRING database (https://string-db.org/) for PPI network construction. The species was designated as “*Homo sapiens*,” with a minimum interaction score of 0.4 to ensure the reliability of the results. Other parameters remained at their default settings. The results were saved in TSV format and imported into Cytoscape 3.7.2 for network analysis (Cytoscape → Tools → NetworkAnalyzer → Analyze Network). The network analysis results were saved, with node size and color reflecting the degree value (larger nodes indicate higher degree values), and edge thickness representing the combined score (thicker edges indicate higher combined scores). Core targets were selected to create a PPI network diagram.

### 2.5. Gene Ontology (GO) Enrichment and Kyoto Encyclopedia of Genes and Genomes (KEGG) pathway analysis

The overlapping genes were uploaded to the DAVID database (DAVID Functional Annotation Bioinformatics Microarray Analysis, https://davidbioinformatics.nih.gov/) for GO enrichment analysis. Gene identifiers were designated as OFFICIAL_GENE_SYMBOL, and the species were identified as *H sapiens*. GO terms were systematically categorized into biological processes (BPs), cellular components (CC), and molecular functions (MF). KEGG pathway enrichment analysis was conducted to pinpoint T2DM-related signaling pathways. The top 10 GO terms across BP, CC, and MF categories, along with the top 18 T2DM-associated KEGG pathways (*P* < .01), were selected to elucidate the therapeutic mechanisms of Sang Huang.

### 2.6. Molecular docking analysis

Molecular docking of Sang Huang active components and key targets was performed using AutoDock Vina (1.1.2; The Scripps Research Institute, Olson Laboratory, La Jolla) to validate their interaction efficacy. Active compounds of Sang Huang were retrieved in SDF format from PubChem and subjected to energy minimization in ChemBio3D, followed by hydrogen addition, charge calculation, rotatable bond assignment, and conversion to PDBQT format via AutoDockTools-1.5.6. Key target proteins (prioritizing human-derived structures with high resolution and ligand structural similarity) were downloaded from the PDB database. Proteins were preprocessed in PyMOL (2.3.0; Schrödinger, LLC, New York) to remove native ligands and water molecules and then processed in AutoDockTools to add hydrogens, assign charges, and save as PDBQT files. Binding sites were predicted using POCASA 1.1, with a grid box size of 60 × 60 × 60 Å (0.375 Å spacing). Molecular docking simulations were performed under default parameters, and interaction patterns between Sang Huang bioactive components and core targets were analyzed using PyMOL 2.3.0. In general, nodes with higher degree centrality hold greater topological importance in a network.^[13]^ Thus, proteins ranked highest by degree centrality in the PPI network are likely to play critical roles in Sang Huang therapeutic effects on T2DM.

### 2.7. Molecular dynamics (MD) simulations

MD simulations were conducted using GROMACS 2021.5. Protein topology parameters were derived from the AMBERGS force field (Garcia & Sanbonmatsu, PNAS 99, 2782–2787, 2002) via the PDB2GMX tool. Ligand topologies were generated with sobtop using the GAFF force field, and hydrogenation was performed in Avogadro. The solvated system (employing the TIP3P water model) was neutralized with Na+/Cl − ions and minimized to eliminate steric clashes. Following equilibration in NVT (0–300 K) and NPT (300 K, 1 bar) ensembles, a 100 ns production MD simulation was conducted. Trajectories were rigorously analyzed for root mean square deviation (RMSD) and root mean square fluctuation (RMSF).

### 2.8. Ethical approval and consent

This study is based solely on in silicoanalysis, including network pharmacology, molecular docking, and molecular dynamics simulations. All data were obtained from publicly available databases. No human participants or animals were involved in the research. Therefore, ethical approval from an institutional review board and informed consent were not required for this study.

## 3. Research results

### 3.1. Active components and target prediction of Sang Huang

Using the BATMAN-TCM database with a score cutoff > 0.84 (LR = 80.88) and *P* < .05, 17 components of Sang Huang were identified, yielding 187 non-redundant drug targets after deduplication.

### 3.2. T2DM-related targets

**Figure 1.**
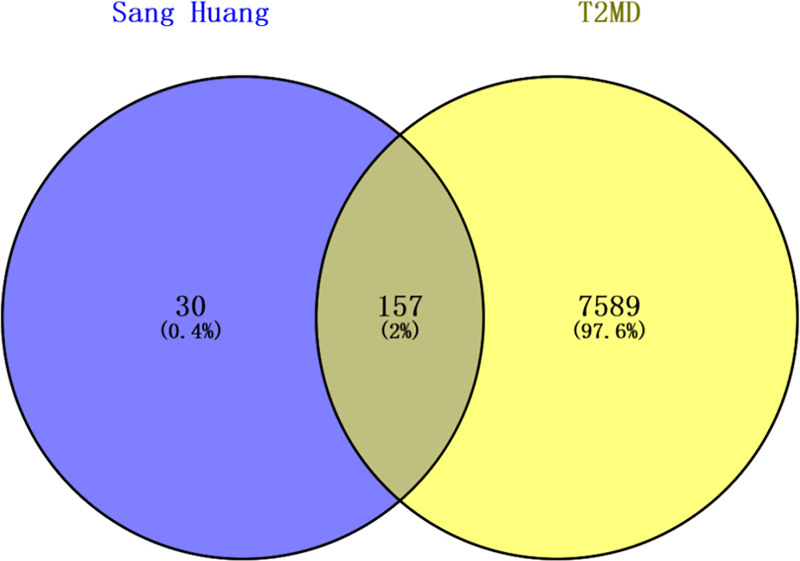
Venn diagram of the overlapping targets between Sang Huang and type 2 diabetes mellitus (T2DM). The left circle represents targets of Sang Huang, and the right circle represents T2DM-related targets. The intersection represents the 157 potential therapeutic targets.

T2DM-associated targets were retrieved from the GeneCards (relevance score > 10) and OMIM databases, resulting in 7348 and 521 targets, respectively. After merging and deduplication, 7746 T2DM-related targets were obtained and standardized via UniProt. Intersection analysis between Sang Huang targets and T2DM targets identified 157 overlapping genes, representing potential therapeutic targets for T2DM (Figure 1, File S1, Supplemental Digital Content, https://links.lww.com/MD/Q409). These are the potential targets of action of T2DM for the treatment of periodontitis.

### 3.3. Drug–component–target network results

**Figure 2.**
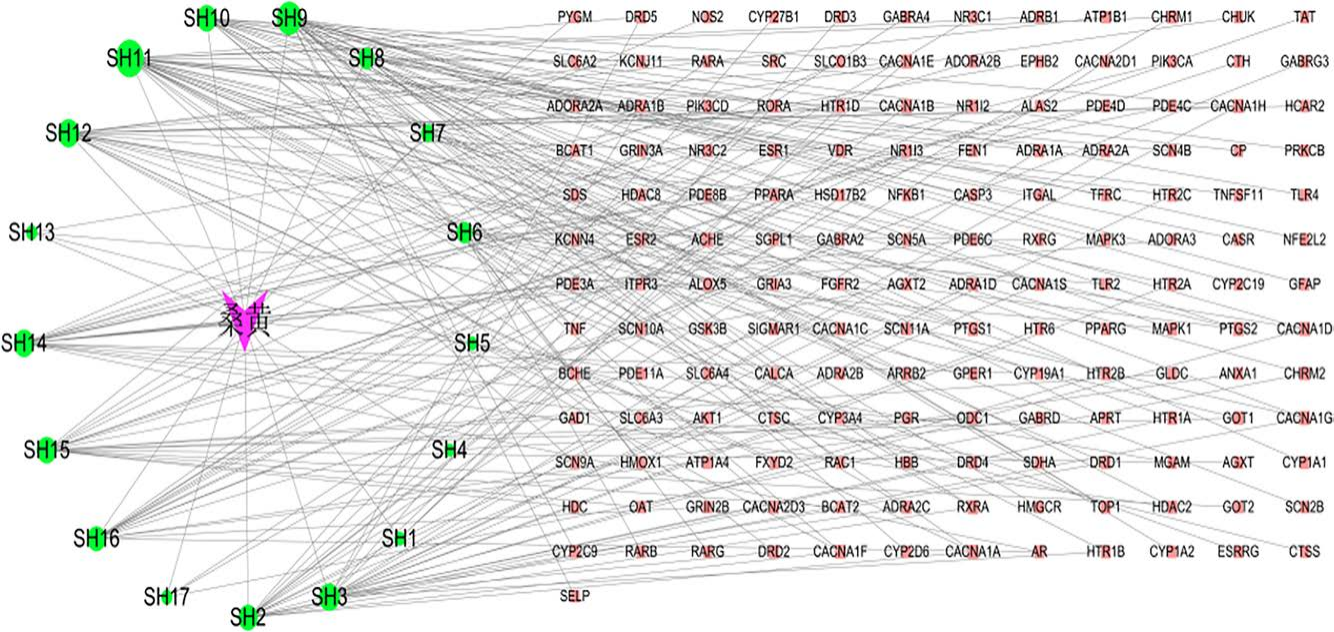
The “drug–component–target” interaction network. Rectangles represent targets, circles represent drug ingredients, and V-shaped nodes represent traditional Chinese medicines. Edges represent interactions between them. CA = caffeic acid; EDP = estradiol dipropionate; ERG = ergotamine.

A “drug–component–target” network was constructed using Cytoscape 3.8.2. As illustrated in Figure 2, we incorporated data from “network.xlsx” and “type.xlsx” files. The network comprised 175 nodes and 231 edges. The top 5 components by degree centrality were ergotamine (ERG; CAS: 113-15-5), caffeic acid (CA; CAS: 331-39-5), estradiol dipropionate (EDP; CAS: 113-38-2), p-hydroxybenzaldehyde-D5 (CAS: 123-08-0), and isoergosterone (CAS: 57-87-4).

### 3.4. Core targets and PPI network

**Figure 3.**
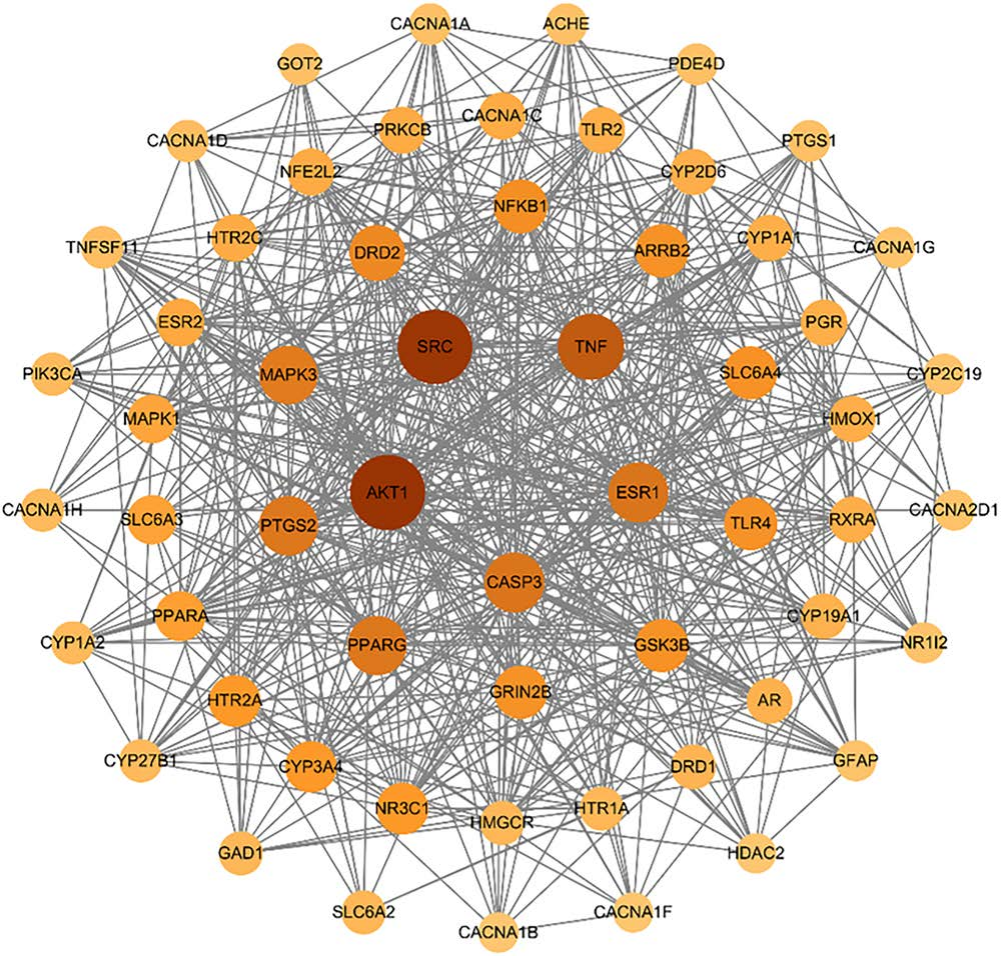
Protein–protein interaction (PPI) network of the common targets between Sang Huang and T2DM. Nodes are colored and sized according to their degree of connectivity. Thicker edges represent higher combined confidence scores. The top 5 hub targets (AKT1, SRC, TNF, ESR1, CASP3) are labeled. AKT1 = v-akt murine thymoma viral oncogene homolog 1, CASP3 = cysteine-dependent aspartate-specific protease-3, ESR1 = estrogen receptor 1, SRC = sarcoma proto-oncogene, T2DM = type 2 diabetes mellitus, TNF = tumor necrosis factor.

The intersection analysis of drug targets and T2DM-associated genes identified 157 overlapping targets, constituting potential therapeutic candidates for Sang Huang-mediated T2DM intervention. To elucidate the molecular interaction network, we systematically uploaded these targets to the STRING database (https://string-db.org/) for PPI prediction. The analysis was conducted using *H sapiens* species-specific data with a confidence score threshold of 0.4 to ensure reliability. The resultant PPI network (TSV format) was subsequently visualized and analyzed using Cytoscape v3.8.2. This robust network comprised 156 nodes and 1264 edges, with topological properties determined through degree centrality measurements. Nodes were proportionally sized and color-coded according to their connectivity strength, whereas edge thicknesses reflected the combined interaction confidence scores. By screening based on the average values of degree, density, and betweenness centrality indicators, 58 core targets were obtained (File S2, Supplemental Digital Content, https://links.lww.com/MD/Q409). The PPI circle diagrams were produced according to the different degree values of the core targets. As illustrated in Figure 3, the top 5 hub proteins identified through this analysis were v-akt murine thymoma viral oncogene homolog 1 (AKT1), sarcoma proto-oncogene (SRC), tumor necrosis factor (TNF), estrogen receptor 1 (ESR1), and cysteine-dependent aspartate-specific protease-3 (CASP3). These proteins demonstrated significant centrality, suggesting their pivotal roles in mediating network dynamics. Crucially, to validate the functional coherence and biological relevance of these overlapping targets, beyond their mere topological centrality, we incorporated functional enrichment analysis (Section 3.5). This analysis unveiled significant clustering within pathways associated with T2DM, notably the PI3K–Akt signaling pathway (*P* < .01), which exhibited high gene enrichment (e.g., AKT1, SRC) and pathway coherence (Fig. 5A and B). Specifically, the PPI network demonstrated a modular organization, where hub proteins such as AKT1 and TNF played context-specific roles in T2DM pathogenesis, rather than merely serving as ubiquitous signaling nodes. AKT1 acted as a regulator of insulin sensitivity through FoxO1 phosphorylation (Section 3.6; molecular docking confirmed EDP-AKT1 binding), while TNF mediated chronic inflammation linked to β-cell dysfunction (supported by KEGG enrichment in TNF signaling pathway). This evidence addresses potential pleiotropic concerns by anchoring hub proteins to disease-specific mechanisms.

**Figure 4.**
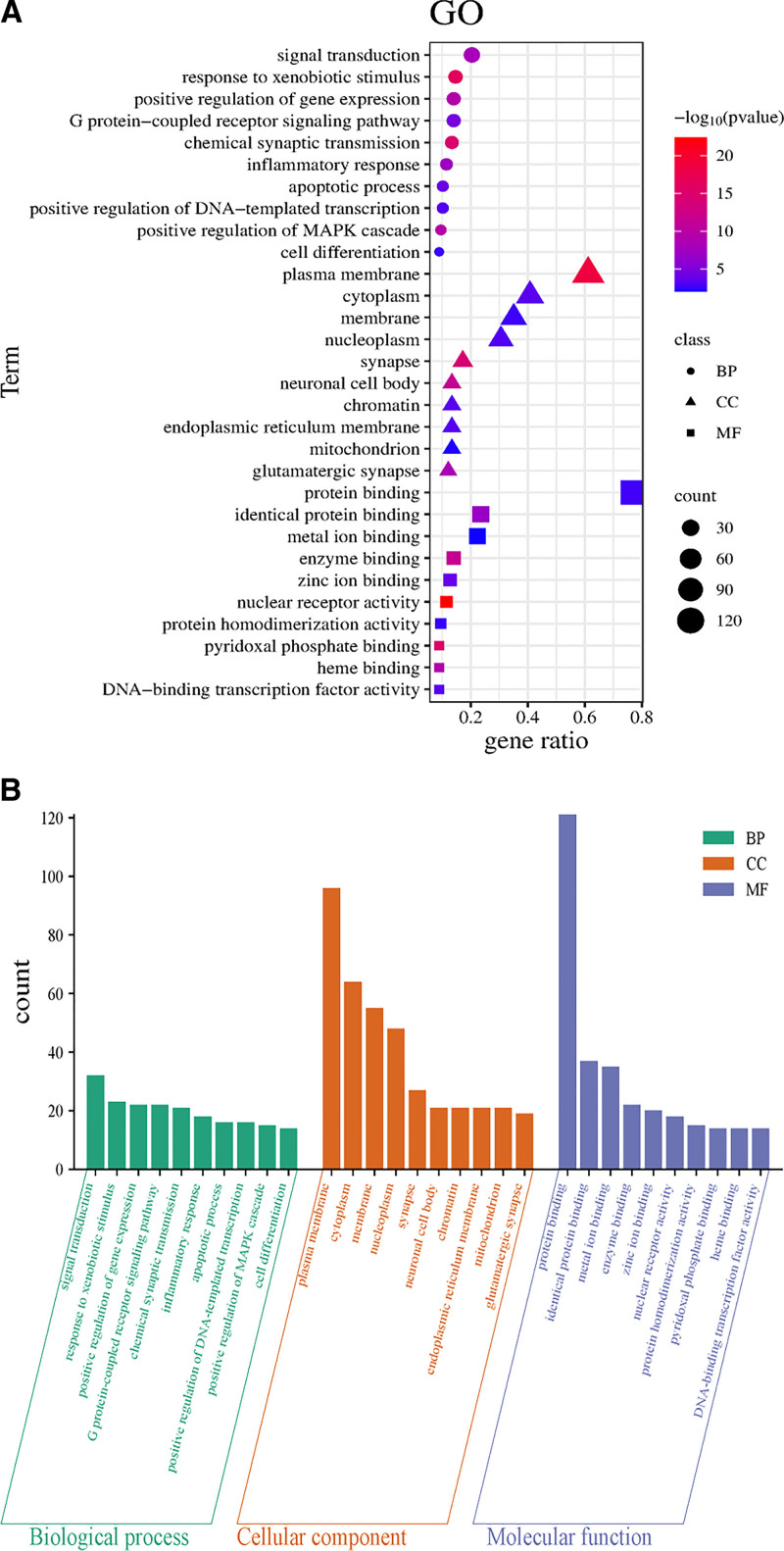
(A) Bubble plot of GO enrichment analysis. (B) Bar graph of GO enrichment analysis (*Note*: The larger the degree value, the larger the shape and the darker the color). GO = Gene Ontology.

### 3.5. Functional enrichment analysis

#### 3.5.1. GO enrichment analysis

GO enrichment analysis of the drug–disease overlapping genes was performed using the DAVID database, identifying 349 GO terms (Fig. 4A and B). Screening with a threshold of *P* < .01 revealed 237 significantly enriched BPs associated with Sang Huang therapeutic effects on T2DM, including signal transduction, response to external stimuli, positive regulation of gene expression, G protein-coupled receptor signaling pathway, chemical synaptic transmission, inflammatory response, apoptotic process, positive regulation of DNA-templated transcription, positive regulation of MAPK cascade, and cell differentiation. CC analysis identified 38 terms, involving the plasma membrane, cytoplasm, membrane, nucleoplasm, neuronal projections, neuronal cell body, chromatin, endoplasmic reticulum membrane, mitochondria, and glutamatergic synapses. MF analysis yielded 74 terms, encompassing protein binding, identical protein binding, metal ion binding, enzyme binding, zinc ion binding, nuclear receptor activity, protein isomerase activity, pyridoxal phosphate binding, heme binding, and DNA-binding transcription factor activity.

#### 3.5.2. KEGG pathway enrichment analysis

**Figure 5.**
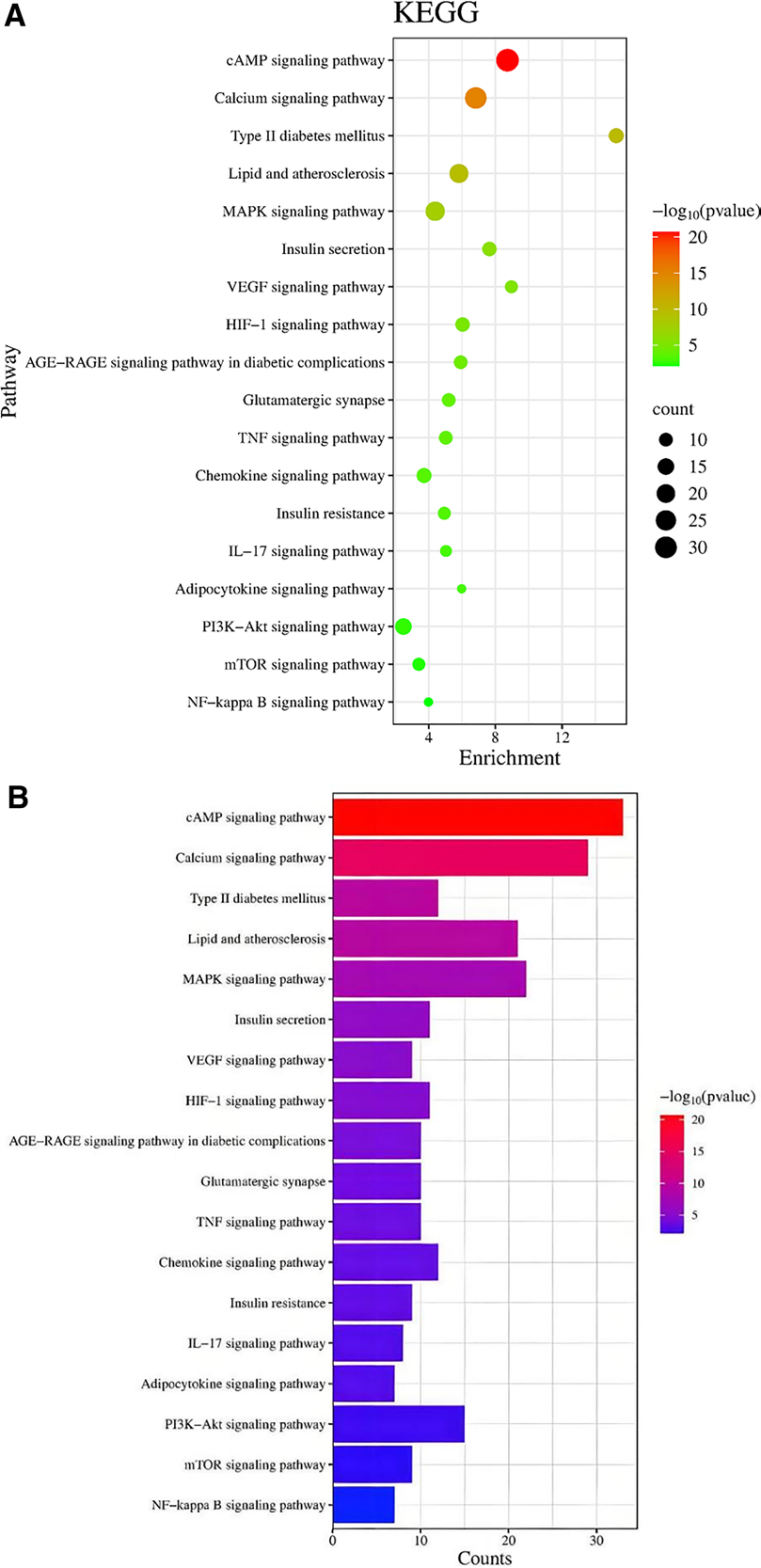
(A) Bubble chart for the same analysis (*Note*: The size of the circle indicates the data of genes enriched in the corresponding pathway, with colors ranging from green to red indicating progressively smaller *P*-values. (B) Bar graph for KEGG pathway enrichment analysis. *P*-value quantifies the probability of observing the experimental results (or more extreme outcomes) under the null hypothesis, with lower values (typically < .05) indicating stronger evidence against the null hypothesis and greater statistical significance. KEGG = Kyoto Encyclopedia of Genes and Genomes.

Pathway enrichment analysis using the DAVID database identified 178 pathways associated with Sang Huang therapeutic effects on T2DM. Screening with a threshold of *P* < .01 yielded 139 significant pathways, from which 18 pathways potentially linked to T2DM were selected (Fig. 5). These pathways were the cAMP signaling pathway, lipid, and atherosclerosis, AGE–RAGE signaling pathway in diabetic complications, insulin resistance, type II diabetes mellitus, adipocytokine signaling pathway, insulin secretion, glutamatergic synapse, TNF signaling pathway, IL-17 signaling pathway, VEGF signaling pathway, PI3K–Akt signaling pathway, mTOR signaling pathway, HIF-1 signaling pathway, NF-kappa B signaling pathway, chemokine signaling pathway, calcium signaling pathway, and MAPK signaling pathway.

From the screened pathways, 18 KEGG metabolic pathways relevant to T2DM were further selected. A bubble chart was subsequently generated based on *P*-values, where the x-axis represents the number of genes enriched in each pathway, bubble size corresponds to the gene count, and color intensity reflects the significance level (−log10 (*P*-value)), providing an intuitive visualization of significantly enriched pathways.

### 3.6. Molecular docking results

**Figure 6.**
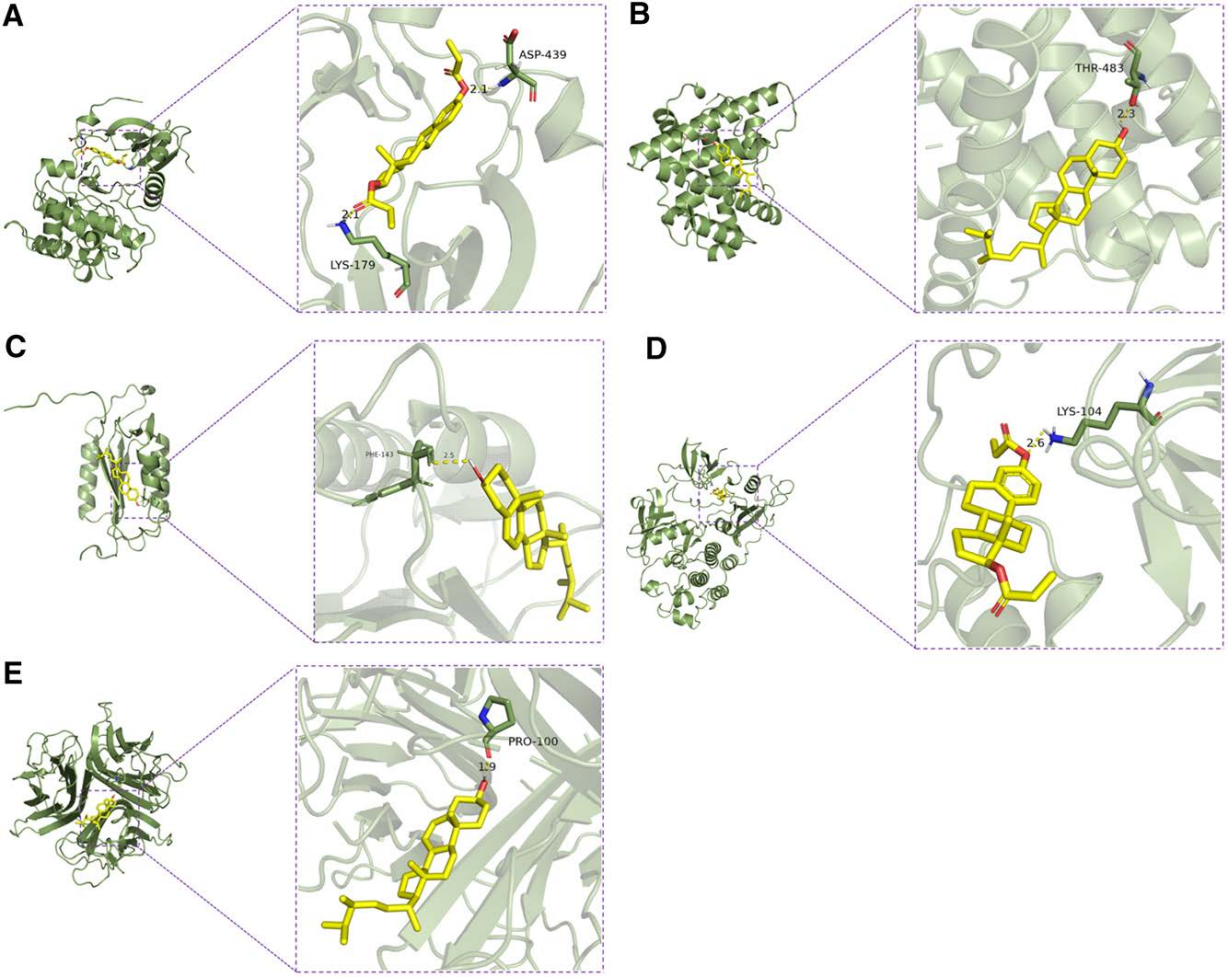
(A) EDP and AKT1 proteins; (B) ERG and ESR1 proteins; (C) EDP and CASP3 proteins; (D) EDP and SRC proteins; (E) ERG and TNF proteins. AKT1 = v-akt murine thymoma viral oncogene homolog 1, CASP3 = cysteine-dependent aspartate-specific protease-3, EDP = estradiol dipropionate, ERG = ergotamine, ESR1 = estrogen receptor 1, SRC = sarcoma proto-oncogene, TNF = tumor necrosis factor.

Molecular docking was performed between the top 5 core targets (AKT1, SRC, TNF, ESR1, and CASP3) and high-degree compounds by semi-flexible docking, with binding affinity (ΔG < 0 indicating spontaneous interaction) as the key metric. As shown in Figure 6, all small molecules successfully occupied the active sites of their respective targets. Key interactions included EDP forming hydrogen bonds with AKT1 (LYS-179 and ASP-439, both 2.1 Å, Fig. 6A), ergosterol bonding to ESR1 (THR-483, 2.3 Å, Fig. 6B), EDP interacting with SRC (LYS-104, 2.6 Å, Fig. 6C), ERG engaging CASP3 (PHE-143, 2.5 Å, Fig. 6D), and ergosterol forming a hydrogen bond with TNF (PRO-100, 1.9 Å, Fig. 6E). These results, detailed in Table 1, demonstrated strong binding potential between Sang Huang bioactive components and critical T2DM-related targets.

**Table 1:** Docking results of core small molecules with core target proteins.

Target	PDB ID	Binding energies of different compounds with targets (kcal/mol)				
		CA	ERG	EDP	Isoergosterone	P-Hydroxybenzaldehyde-D5
TNF	2E7A	-6.7	-9.4	-8.8	-8	-5.2
CASP3	5IC4	-5.2	-7.3	-6.9	-7.0	-4.0
AKT1	6NPZ	-6.8	-8.2	-9	-7.5	-5.2
SRC	2SRC	-6.7	-8.7	-8.2	-7.5	-5.7
ESR1	6SBO	-6.7	-8.7	-8.2	-7.5	-5.7

AKT1 = v-akt murine thymoma viral oncogene homolog 1, CASP3 = cysteine-dependent aspartate-specific protease-3, ESR1 = estrogen receptor 1, SRC = sarcoma proto-oncogene, TNF = tumor necrosis factor.

### 3.7. MD simulation results

To evaluate dynamic stability, we conducted 100 ns MD simulations on 5 complexes (AKT1–EDP, ESR1–ERG, CASP3–ERG, SRC–ERG, and TNF–ERG), focusing on RMSD and RMSF. RMSD quantifies structural deviation from the initial conformation, with lower values indicating greater stability,^[14]^ whereas RMSF reflects residue flexibility, where higher values suggest dynamic regions (e.g., flexible loops) and lower values denote rigid structural motifs (e.g., α-helices).^[15]^

#### 3.7.1. Complex stability analysis

**Figure 7.**
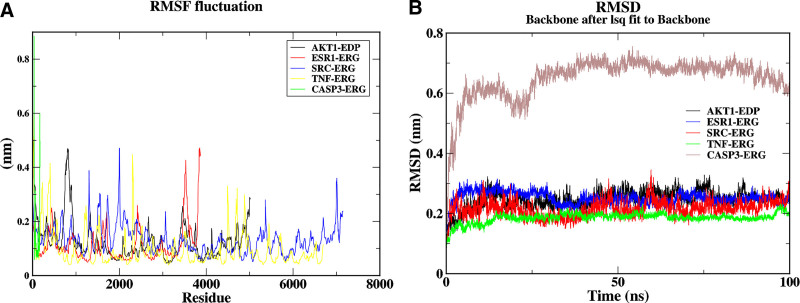
(A) RMSF diagram based on molecular dynamics simulation. (B) RMSD plot based on molecular dynamics simulation. RMSD = root mean square deviation, RMSF = root mean square fluctuation.

To investigate the dynamic properties of the docked complexes, we performed 100 ns MD simulations on 5 complexes (AKT1–EDP, ESR1–ERG, CASP3–ERG, SRC–ERG, and TNF–ERG) based on covalent docking results, focusing on RMSD and RMSF. As shown in Figure 7A, RMSF analysis revealed varying degrees of conformational fluctuations across all complexes at the atomic level (2000–8000 atoms). Notably, the TNF–ERG and CASP3–ERG complex exhibited significantly higher RMSF values compared with the others (e.g., AKT1–EDP and CASP3–ERG), indicating high structural flexibility likely associated with dynamic regulation of functional domains. SRC–ERG and ESR1–ERG displayed similar fluctuation patterns, whereas RMSD analysis (Fig. 7B) demonstrated that the TNF–ERG complex exhibited the lowest and most stable RMSD values (0.15–0.20 nm), surpassing the structural stability of the 4 other systems. The AKT1–EDP complex exhibited significant initial fluctuations (0.20–0.35 nm) but stabilized after 70 ns. Similarly, the ESR1–ERG complex showed moderate RMSD oscillations (0.20–0.30 nm) during the early and mid-stages, stabilizing after 70 ns. The CASP3–ERG complex displayed instability in the mid-simulation phase (rising RMSD values) but gradually equilibrated after 70 ns. The SRC–ERG complex mirrored the stability trend of ESR1–ERG, achieving equilibrium by the end of the simulation. Although the AKT1–EDP system experienced pronounced initial fluctuations, all complexes ultimately stabilized, confirming their structural integrity during interactions.

## 4. Discussion

In recent years, the integration of network pharmacology with metabolomics has revealed multi-target mechanisms of traditional medicines in practical applications, such as Lianhua Qingwen inhibition of COVID-19 viral replication (ACE2 and 3CLpro) and immune modulation (IL-6 and TNF-α).^[16]^ AI-driven screening identified celastrol as an NF-κB pathway inhibitor to enhance PD-1 inhibitor efficacy in melanoma, whereas TNF-α inhibitors combined with probiotics ameliorate obesity-related insulin resistance.^[17]^ These successes highlight the modernization of traditional medicine via the integration of bioinformatics and network pharmacology.

In this study, network pharmacology identified 17 bioactive components of Sang Huang via the BATMAN-TCM database. The top 5 components by degree centrality (ERG, CA, EDP, p-hydroxybenzaldehyde-D5, and isoergosterone) were prioritized for analysis. CA (3,4-dihydroxycinnamic acid), a phenolic acid with antioxidative properties, stimulates insulin secretion in pancreatic β-cells, effectively mitigating postprandial hyperglycemia in T2DM.^[18]^ EDP, a semi-synthetic estrogen, enhances GLUT4 expression via the PKC and AMPK pathways, promoting glucose uptake in L6 skeletal muscle cells through Ca²⁺-dependent mechanisms. Estradiol can inhibit the production of crucial pro-inflammatory cytokines by immune cells such as monocytes/macrophages and dendritic cells, for instance, IL-1β. The impacts of IL-1β on T2DM include the induction of islet cell damage. Both T2DM patients and obese mice exhibit varying degrees of macrophage infiltration in their islets. The IL-1β receptor is highly expressed on the surfaces of islet β-cells and infiltrating macrophages. Upon binding with IL-1β, the receptor activates the NF-κB pathway, further promoting the production of IL-1β, thereby expanding the scope of inflammation, causing dysfunction and damage to islet β-cells, and ultimately leading to reduced insulin secretion.^[19]^ p-Hydroxybenzaldehyde-D5, a deuterated derivative, shows theoretical antioxidative benefits for T2DM-related oxidative stress, but empirical evidence remains lacking.

The PPI network analysis identified 5 core targets of Sang Huang in T2DM intervention: AKT1, SRC, TNF, CASP3, and ESR1, which synergistically regulate inflammatory responses, insulin signaling, and glucose–lipid metabolism. TNF-α, a crucial pro-inflammatory mediator, is aberrantly overexpressed in the adipose tissues of patients with obesity and T2DM. It induces insulin resistance by promoting IRS-1 serine phosphorylation (Ser307) to inhibit insulin receptor signaling and downregulating AKT1 phosphorylation at Thr308/Ser473, thereby impairing GLUT4 membrane translocation and skeletal muscle glucose uptake.^[20–23]^ One of the core pathological characteristics of T2DM is the progressive decline in function and reduction in number of pancreatic beta cells. A crucial mechanism leading to the decrease in beta cell count is apoptosis. In 2010, Stienstra et al identified the significant role of caspase-1 in adipose tissue metabolism and suggested that inhibiting caspase-1 activity may contribute to improving adipocyte metabolism and enhancing insulin sensitivity, thus serving as a potential treatment for obesity and T2DM.^[24]^ The NLRP3 inflammasome leads to the activation of caspase-1 and processes the inactive IL-1β precursor into mature, active IL-1β. Once released, IL-1b can amplify its signals by self-activation via engagement of the IL-1 receptor I leading to a vicious cycle of inflammation.^[4]^ Therefore, we hypothesize that regulating caspase-3 activity may protect beta cells, thereby serving as a therapeutic approach for T2DM. SRC, a critical coactivator of ERα, downregulates lipolysis-related genes (e.g., ATGL and HSL) upon deficiency, leading to lipid accumulation and obesity. ESR1, beyond estrogen metabolism regulation, enhances adipocyte insulin sensitivity via the PPARγ/adiponectin pathway and inhibits TGF-β1-mediated renal fibrosis in diabetic nephropathy.^[25,26]^ AKT1, central to the PI3K–Akt pathway, restores glucose homeostasis by promoting FoxO1 nuclear export, suppressing GSK3β activity, and coordinating hepatic glucose synthesis with muscle uptake.^[27]^

Sang Huang therapeutic effects involve multi-pathway crosstalk. The AGE–RAGE axis reduces diabetic vascular calcification and glomerular basement membrane thickening by blocking NF-κB nuclear translocation (reducing IL-6 and TNF-α) and preventing MAPK/PI3K–Akt-mTOR hyperactivation, while upregulating SOD-1 to alleviate oxidative stress.^[28–32]^ The HIF-1 pathway, under adipose tissue hypoxia, suppresses HIF-1α-driven SOCS3 transcription, thereby relieving SOCS3-mediated inhibition of IRS-1/2 tyrosine phosphorylation (Tyr612) and enhancing adiponectin oligomerization for insulin sensitization.^[33,34]^ cAMP signaling amplifies glucose-dependent insulin secretion in β-cells via GLP-1 receptor-activated cAMP-PKA/EPAC2 cascades, which promote calcium influx and insulin vesicle exocytosis but inhibit apoptosis.^[35,36]^ In the liver, dual-target agonists suppress glucagon-driven pathological gluconeogenesis (e.g., PEPCK) by modulating cAMP intensity.^[37]^ The PI3K–Akt cascade activates PDK1-dependent AKT phosphorylation to enhance mTORC1-mediated β-cell proliferation, inhibit FoxO1-regulated gluconeogenic genes (PEPCC and G6Pase), and amplify GSK3β-driven glycogen synthase activation.^[27]^ Notably, crosstalk between the MAPK and PI3K–Akt pathways in GLUT4 vesicle trafficking suggests that SRC kinase integrates these networks to enhance insulin’s metabolic effects.

GO enrichment analysis validated these mechanisms at the systems biology level. Enriched BPs (e.g., inflammatory response, MAPK cascade regulation, and G protein-coupled receptor signaling) align with the chronic low-grade inflammation and insulin secretory defects of T2DM. CC terms designated targets to insulin action hubs: the plasma membrane (GLUT4 trafficking complexes), mitochondrial inner membrane (oxidative phosphorylation), and endoplasmic reticulum (unfolded protein response). Molecular functions encompassed nuclear receptor binding, kinase adaptor activity, and DNA-binding transcription factor coactivation.

Sang Huang exhibits multi-target, multi-pathway therapeutic potential for T2DM, modulating insulin signaling, oxidative stress, and inflammatory responses through bioactive components like CA and EDP. Its core targets (AKT1, TNF) and associated pathways (AGE–RAGE and PI3K–Akt) provide a molecular basis for further preclinical and clinical validation.

While the PPI network constructed from the STRING database provides valuable insights into the potential interactions among Sang Huang targets, it is important to acknowledge the inherent limitations of such high-throughput data. The STRING database integrates both experimentally validated and computationally predicted interactions, which, if not carefully filtered and interpreted, could introduce false positives and inflate the network.^[38]^ To mitigate this concern and enhance the biological relevance of our findings, we implemented a multi-tiered strategy: first, we applied a conservative confidence score threshold (≥0.4) during network construction to prioritize higher-quality interactions. More importantly, we did not rely on topological features (e.g., hub proteins) alone. The functional coherence of the network was rigorously tested through subsequent GO and KEGG enrichment analyses (Section 3.5). The significant enrichment of these 157 overlapping targets in biologically coherent pathways closely related to T2DM pathogenesis (such as the PI3K–Akt signaling pathway and TNF signaling pathway) strongly suggests that the observed PPI network is not a random assemblage but reflects a meaningful functional module underlying the therapeutic effect of Sang Huang. Furthermore, the practical biological significance of key hub targets (e.g., AKT1, TNF) was further corroborated by molecular docking results (Section 3.6), which demonstrated strong binding affinities between Sang Huang active compounds and these proteins. This convergence of network pharmacology prediction, functional enrichment, and computational validation helps to counterbalance potential noise from the PPI data and strengthens the credibility of our core target conclusions.

The present study has the following limitations: although molecular docking and dynamic simulations suggest the stability of interactions such as ERG–TNF and EDP–AKT1, they lack in vitro binding experiments and functional validation at the cellular level. The bioavailability of components has not been considered, for example, the bioactivity of CA significantly decreases after it is metabolized into hippuric acid in the intestine,^[39]^ and its actual in vivo effects may deviate from predictions. Additionally, the regulatory effects of *Phellinus linteus* extract on core pathways and its glucose metabolism-improving effects have not been validated in T2DM animals.

## 5. Conclusion

Molecular docking and dynamics simulations confirmed the stable binding of Sang Huang components (e.g., EDP and ERG) to TNF and AKT1, supporting their role in T2DM intervention. The mechanism of action of Sang Huang in the treatment of T2DM exhibits characteristics of multiple components, multiple targets, and multiple pathways. We speculate that TNF, AKT1, SRC, CASP3, and ESR1 are key targets, and signaling pathways such as PI3K–Akt are crucial. However, further experimental validation is required.

## Author contributions

**Conceptualization:** Deqiang Zhu.

**Data curation:** Ke Tang.

**Formal analysis:** Haiyan Fang, JingZhen Wang, Xinli Liu.

**Funding acquisition:** Deqiang Zhu.

**Investigation:** Hao Li, Ziyu Zheng, Gengyao Zhen.

**Methodology:** Haiyan Fang, JingZhen Wang, Xinli Liu.

**Software:** Ke Tang.

**Validation:** Hao Li, Ziyu Zheng, Gengyao Zhen.

**Writing – original draft:** Hao Li, Ziyu Zheng, Gengyao Zhen.

**Writing – review & editing:** Deqiang Zhu.

## Supplementary Material



## References

[1] DeFronzo RA Ferrannini E Groop L. Type 2 diabetes mellitus. Nat Rev Dis Primers. 2015;1:15019. 27189025 10.1038/nrdp.2015.19

[2] Guo W Song Y Sun Y. Systemic immune-inflammation index is associated with diabetic kidney disease in type 2 diabetes mellitus patients: evidence from NHANES 2011-2018. Front Endocrinol. 2022;13:1071465. 10.3389/fendo.2022.1071465 PMC9763451 36561561

[3] Einarson TR Acs A Ludwig C Panton UH. Prevalence of cardiovascular disease in type 2 diabetes: a systematic literature review of scientific evidence from across the world in 2007–2017. Cardiovasc Diabetol. 2018;17:83. 29884191 10.1186/s12933-018-0728-6 PMC5994068

[4] Dinarello CA Donath MY Mandrup-Poulsen T. Role of IL-1β in type 2 diabetes. Curr Opin Endocrinol Diabetes Obes. 2010;17:314–21. 20588114 10.1097/MED.0b013e32833bf6dc

[5] González P Lozano P Ros G Solano F. Hyperglycemia and oxidative stress: an integral, updated and critical overview of their metabolic interconnections. Int J Mol Sci . 2023;24:9352. 37298303 10.3390/ijms24119352 PMC10253853

[6] Ferrari R Catapano AL. Residual cardiovascular risk. Eur Heart J Suppl. 2016;18(suppl C):C1–C1. 28533704 10.1093/eurheartj/suw010

[7] Ma HT Hsieh JF Chen ST. Anti-diabetic effects of ganoderma lucidum. Phytochem. 2015;114:109–13. 10.1016/j.phytochem.2015.02.017 25790910

[8] Lee SK Ryu SH Turk A. Characterization of α-glucosidase inhibitory constituents of the fruiting body of lion’s mane mushroom (hericium erinaceus). J Ethnopharmacol. 2020;262:113197. 32738392 10.1016/j.jep.2020.113197

[9] Xiao H Li H Wen Y. Tremella fuciformis polysaccharides ameliorated ulcerative colitis via inhibiting inflammation and enhancing intestinal epithelial barrier function. Int J Biol Macromol. 2021;180:633–42. 33744251 10.1016/j.ijbiomac.2021.03.083

[10] Zhu L Song J Zhou JL Si J Cui BK. Species diversity, phylogeny, divergence time, and biogeography of the genus sanghuangporus (basidiomycota). Front Microbiol. 2019;10:812. 31057518 10.3389/fmicb.2019.00812 PMC6478708

[11] Chen H Tian T Miao H Zhao YY. Traditional uses, fermentation, phytochemistry and pharmacology of phellinus linteus: a review. Fitoterapia. 2016;113:6–26. 27343366 10.1016/j.fitote.2016.06.009

[12] Kern PA Saghizadeh M Ong JM Bosch RJ Deem R Simsolo RB. The expression of tumor necrosis factor in human adipose tissue. Regulation by obesity, weight loss, and relationship to lipoprotein lipase. J Clin Invest. 1995;95:2111–9. 7738178 10.1172/JCI117899 PMC295809

[13] Freeman LC. Centrality in social networks conceptual clarification. Soc Netw. 1978;1:215–39.

[14] Brüschweiler R. Efficient RMSD measures for the comparison of two molecular ensembles. Proteins Struct Funct Bioinforma. 2003;50:26–34. 10.1002/prot.10250 12471596

[15] Vázquez SA Otero XL Martinez-Nunez E. A trajectory-based method to explore reaction mechanisms. Molecules. 2018;23:3156. 30513663 10.3390/molecules23123156 PMC6321347

[16] Xia Q Xun Y Lu J. Network pharmacology and molecular docking analyses on lianhua qingwen capsule indicate Akt1 is a potential target to treat and prevent COVID-19. Cell Prolif. 2020;53:e12949. 33140889 10.1111/cpr.12949 PMC7705900

[17] Depommier C Everard A Druart C. Supplementation with akkermansia muciniphila in overweight and obese human volunteers: a proof-of-concept exploratory study. Nat Med. 2019;25:1096–103. 31263284 10.1038/s41591-019-0495-2 PMC6699990

[18] Eid HM Thong F Nachar A Haddad PS. Caffeic acid methyl and ethyl esters exert potential antidiabetic effects on glucose and lipid metabolism in cultured murine insulin-sensitive cells through mechanisms implicating activation of AMPK. Pharm Biol. 2017;55:2026–34. 28832228 10.1080/13880209.2017.1345952 PMC6130489

[19] Alexandraki K Piperi C Kalofoutis C Singh J Alaveras A Kalofoutis A. Inflammatory process in type 2 diabetes: the role of cytokines. Ann N Y Acad Sci. 2006;1084:89–117. 17151295 10.1196/annals.1372.039

[20] Hotamisligil GS Arner P Caro JF Atkinson RL Spiegelman BM. Increased adipose tissue expression of tumor necrosis factor-alpha in human obesity and insulin resistance. J Clin Invest. 1995;95:2409–15. 7738205 10.1172/JCI117936 PMC295872

[21] Saghizadeh M Ong JM Garvey WT Henry RR Kern PA. The expression of TNF alpha by human muscle. Relationship to insulin resistance. J Clin Invest. 1996;97:1111–6. 8613535 10.1172/JCI118504 PMC507159

[22] Son M Wu J. Egg white hydrolysate and peptide reverse insulin resistance associated with tumor necrosis factor-α (TNF-α) stimulated mitogen-activated protein kinase (MAPK) pathway in skeletal muscle cells. Eur J Nutr. 2019;58:1961–9. 29955954 10.1007/s00394-018-1753-7 PMC6647935

[23] Uysal KT Wiesbrock SM Marino MW, Hotamisligil GS. Protection from obesity-induced insulin resistance in mice lacking TNF-alpha function. Nature. 1997;389:610–4. 9335502 10.1038/39335

[24] Stienstra R Joosten LA Koenen T. The inflammasome-mediated caspase-1 activation controls adipocyte differentiation and insulin sensitivity. Cell Metab. 2010;12:593–605. 21109192 10.1016/j.cmet.2010.11.011 PMC3683568

[25] Dagar N Jadhav HR Gaikwad AB. Network pharmacology combined with molecular docking and dynamics to assess the synergism of esculetin and phloretin against acute kidney injury-diabetes comorbidity. Mol Divers. 2025;29:1–19. 38578376 10.1007/s11030-024-10829-5

[26] Zhao WM Wang ZJ Shi R Zhu Y Li XL Wang DG. Analysis of the potential biological mechanisms of diosmin against renal fibrosis based on network pharmacology and molecular docking approach. BMC Complement Med Ther. 2023;23:157. 37179298 10.1186/s12906-023-03976-z PMC10182711

[27] Mauvais-Jarvis F Clegg DJ Hevener AL. The role of estrogens in control of energy balance and glucose homeostasis. Endocr Rev. 2013;34:309–38. 23460719 10.1210/er.2012-1055 PMC3660717

[28] Daroux M Prévost G Maillard-Lefebvre H. Advanced glycation end-products: implications for diabetic and non-diabetic nephropathies. Diabetes Metab. 2010;36:1–10. 19932633 10.1016/j.diabet.2009.06.005

[29] Abel M, Ritthaler U, Zhang Y, et al. Expression of receptors for advanced glycosylated end-products in renal disease. Nephrol Dial Transplant. 1995;10:1662–7. 8559486

[30] Hou FF Ren H Owen WF. Enhanced expression of receptor for advanced glycation end products in chronic kidney disease. J Am Soc Nephrol. 2004;15:1889–96. 15213278 10.1097/01.asn.0000131526.99506.f7

[31] Kay AM Simpson CL Stewart JA. The role of AGE/RAGE signaling in diabetes-mediated vascular calcification. J Diabetes Res. 2016;2016:1–8. 10.1155/2016/6809703 PMC4980539 27547766

[32] Suzuki D Toyoda M Yamamoto N. Relationship between the expression of advanced glycation end-products (AGE) and the receptor for AGE (RAGE) mRNA in diabetic nephropathy. Intern Med. 2006;45:435–41. 16679697 10.2169/internalmedicine.45.1557

[33] Gonzalez FJ Xie C Jiang C. The role of hypoxia-inducible factors in metabolic diseases. Nat Rev Endocrinol. 2019;15:21–32. 10.1038/s41574-018-0096-z PMC6624429 30275460

[34] Jiang C Kim JH Li F. Hypoxia-inducible factor 1α regulates a SOCS3-STAT3-adiponectin signal transduction pathway in adipocytes. J Biol Chem. 2013;288:3844–57. 23255598 10.1074/jbc.M112.426338 PMC3567639

[35] Drucker DJ. Mechanisms of action and therapeutic application of glucagon-like peptide-1. Cell Metab. 2018;27:740–56. 29617641 10.1016/j.cmet.2018.03.001

[36] Holst JJ. The physiology of glucagon-like peptide 1. Physiol Rev. 2007;87:1409–39. 17928588 10.1152/physrev.00034.2006

[37] Cypess AM Weiner LS Roberts-Toler C. Activation of human brown adipose tissue by a β3-adrenergic receptor agonist. Cell Metab. 2015;21:33–8. 25565203 10.1016/j.cmet.2014.12.009 PMC4298351

[38] Szklarczyk D Gable AL Nastou KC. The STRING database in 2021: customizable protein–protein networks, and functional characterization of user-uploaded gene/measurement sets. Nucleic Acids Res. 2021;49:D605–12. 33237311 10.1093/nar/gkaa1074 PMC7779004

[39] Olthof MR Hollman PC Katan MB. Chlorogenic acid and caffeic acid are absorbed in humans. J Nutr. 2001;131:66–71. 11208940 10.1093/jn/131.1.66

